# Plasma-Corona Enabled Synthesis of Photonic Copper Sensor for the Detection of Ovarian Cancer Marker CA 125

**DOI:** 10.3390/nano16140894

**Published:** 2026-07-21

**Authors:** Kimberly M. Jones, Takumi Uesaka, Lakshmi V. Nair, Vinoy Thomas

**Affiliations:** 1 Department of Mechanical and Materials Engineering, School of Engineering, University of Alabama at Birmingham, Birmingham, AL 35294, USAtuesaka@uab.edu (T.U.); lakshmi.vnair@vit.ac.in (L.V.N.); 2 Centre for NanoBiotechnology, Vellore Institute of Technology, Vellore 632014, Tamil Nadu, India; 3 Center for Nanoscale Materials and Biointegration (CNMB), University of Alabama at Birmingham, Birmingham, AL 35294, USA; 4 Center for Clinical and Translational Sciences (CCTS), University of Alabama at Birmingham, Birmingham, AL 35294, USA

**Keywords:** low temperature plasma, sensors, fluorescence, biomarkers, cancer detection, metal nanoclusters, copper nanoparticles

## Abstract

The objective of this research is the development of a copper-based optical sensor for the detection of ovarian cancer marker CA 125 synthesized using low-temperature plasma. Optical materials produced with metals show unique advantages due to their ability to interact with light. There are different methods currently used for the synthesis of optical materials that can be associated with longer processing times and low material yield. The novelty of this study is the development of copper-based optical material (CuPy) using low-temperature plasma and subsequent modification for the detection of CA 125. **Introduction:** Plasma consists of a mixture of fully and partially ionized gas. It comprises diverse, highly energized species of atoms, ions, electrons, excited molecules, and charged species. These energized species are used to create new materials, for surface modifications, and in medical applications. Plasma can create a controlled environment for the creation of novel materials. Using low-temperature plasma, it will be possible to have precise control of the chemical composition and structure due to the creation of excited molecules, ions, and free radicals. **Method:** The CuPy material was synthesized using radio-frequency-assisted low-temperature plasma. Prior to synthesis, the plasma chamber was cleaned using radio frequency (RF) plasma without any reagents or gases. RF plasma was used for the synthesis of CuPy for 10 min and subsequent hydrogen plasma (50 sccm) for another 10 min. Two types of products were extracted from the chamber (one in water and another in methanol). These two products were analyzed using UV–visible absorbance spectroscopy, fluorescence spectroscopy, X-ray photoelectron spectroscopy (XPS), and Fourier transform infrared spectroscopy (FTIR). The methanol extracted samples were further modified with CA 125 antibody. Zeta potential measurements were performed to confirm the binding of the CA 125 antibody to the sensor. The sensing efficacy of the sensor towards CA 125 antigen was monitored using fluorescence spectroscopy. **Results:** The absorbance spectrum of methanol extracted CuPy shows absorbances around 251 nm, 282 nm, and 339 nm. The extracted product exhibited a red edge excitation emission in the visible region. The elemental composition and oxidation state of the sample were evaluated using XPS. CA 125 antibody conjugation with CuPy was confirmed using UV–visible absorbance spectroscopy, fluorescence spectroscopy, and FTIR spectroscopy. The antibody binding resulted in the fluorescence shifts towards higher wavelengths with an increase in the emission intensity compared with CuPy. Zeta potential measurements also confirmed the binding of the CA 125 antibody to the sensor. Different concentrations of CA 125 antigen resulted in the quenching of fluorescence. This change in the fluorescence intensity was used for the detection of CA 125. **Conclusions:** A copper-based optical material was developed using low-temperature plasma, and it was found to be effective for the detection of CA 125 ovarian cancer marker.

## 1. Introduction

Plasma, which is considered the fourth state of matter, consists of a mixture of partially ionized gas. It comprises diverse, highly energized species of atoms, ions, electrons, excited molecules, and charged species. These energized species can be used to create new materials, for surface modifications, and in medical applications [1,2,3,4,5,6,7,8]. Plasma can be used as a controlled environment for the creation of novel materials. Using nonthermal low-temperature plasma (LTP), it is possible to have precise control of the chemical composition and structure due to the creation of excited molecules, ions, and free radicals. This technique allows materials science to fabricate functional materials with enhanced properties such as optical properties, electrical properties, conductivity, durability, etc. [9,10,11]. The plasma-assisted method is also one of the material processing techniques used in metallurgy, aerospace, and the semiconductor industry. This technique, especially low-temperature plasma, is pollution-free and energy saving when compared to other techniques [6]. LTP-assisted materials are widely used in energy, medical, and environmental applications [12,13,14,15,16,17].

Due to light absorption and emission properties, optical materials are among the promising materials in the field of bioimaging and biomolecule sensing [18,19,20,21,22,23]. These materials have the ability to interact with light in the visible to near infrared region imparting high specificity and selectivity [24]. These materials are the major components of optical fibers, photonic crystal, and light sensors which can detect and analyze changes in the light property including wavelength, intensity, energy, etc. Optical materials offer nondestructive, reliable, accurate, and real-time monitoring of target analytes and tissues [25,26,27]. The optical method of detection of the analyte is more efficient when compared to other conventional methods [28,29,30].

The advantages of optical materials in the field of diagnostics are their sensitivity and versatility for functionalization towards target analytes [31,32,33,34,35,36]. These materials can be easily engineered or manipulated in such a way that interactions with specific analytes result in variations to their optical properties even in smaller quantities. For example, these materials, upon interacting with body fluids or biomarkers, exhibit changes in their optical properties which are used for the detection of cancer and other medical conditions. These materials are found to be selective and sensitive biosensors with greater efficacy and reliability. Even small amounts of biological analytes could be measured with the aid of such sensors [37,38,39,40]. Additionally, optical sensors allow for the miniaturization of these devices and make them useful for developing portable point-of-care diagnostic devices. These sensor devices are efficient, accurate, and cost-effective solutions for the healthcare sector [41,42].

Cancer antigen 125 (CA 125) is a glycoprotein biomarker used for the monitoring and diagnosis of ovarian cancer. This glycoprotein is found in blood and produced by the ovarian cells and other tissues such as endometrium and peritoneum. An elevated level of CA 125 is associated with ovarian cancer. An elevated level of CA 125 is also associated with endometriosis, pelvic inflammatory disease, and liver disease [43,44,45,46,47]. CA 125 is widely used as a marker for the early detection of ovarian cancer and for monitoring cancer progression, treatment response, and its recurrence. Due to the possibility of false negative results in clinical diagnostics, ovarian cancer diagnoses are confirmed along with other diagnostic techniques [43].

The early detection of elevated levels of CA 125 is one of the promising diagnostic tools for the proper management of ovarian cancer. This cancer is usually detected at a later stage when the treatment options are limited. It is fifth leading cause of cancer-related deaths in women. The early symptoms of the disease are subtle and can result in a delay in the diagnosis. Accurate and effective monitoring of the CA 125 level will be a valuable marker for the early intervention of ovarian cancer and its further follow up. Regular monitoring of the CA 125 level is a good option for those patients who are undergoing treatment to assess the usefulness of the therapy. This monitoring will help the physician make personalized and timely treatment decisions. Therefore, developing sensitive and accurate methods for CA 125 detection is crucial for improving patient outcomes in ovarian cancer management. Optical materials have been explored as sensors for the detection of CA 125 [48]. A novel optical biosensor for CA 125 was developed using nanogold coated with a sol–gel matrix. CA 125 analyte binding resulted in the fluorescence quenching at 423 nm upon excitation at 340 nm [49]. Other optical materials used for the detection of CA 125 include carbon quantum dots, oil-based organo-hydrogels, n-type nanowire biosensors, etc. [50,51,52].

Optical materials composed of metals, such as gold, silver, and copper, show unique advantages due to their ability to interact with light. Metals can produce surface plasmon which enhances the interaction of light with matter and makes them ideal candidates in various applications. Metal-based optical sensors are widely explored for the detection of chemicals and biomolecules at very low concentrations [53,54,55,56,57,58,59]. Metal-based optical sensors can be used for label-free detection which helps to eliminate the need to chemically alter the analyte molecule [60]. The wet chemical method using metal precursors and desired ligands is the easiest route employed for the synthesis of these materials. However, this method is often associated with multiple byproducts and unreacted precursors. Washing is one of the crucial steps in this method, and the material yield obtained in this process is very low. As a result, scalability is more difficult which limits its applications on the commercial level. In contrast, plasma is known for its scalability and versatility.

The synthesis of metallic materials using LTP is often challenging due to their high melting point. To address this issue, this study used pyrrole, a heterocyclic aromatic organic compound, along with a metal precursor. Among the optical materials, copper is widely explored compared to gold and silver due to its low cost and abundance [61,62,63,64,65,66,67,68,69]. The optical property of copper (Cu) can be easily manipulated due to its varied oxidation states. Optical materials using metals were explored as a sensor for various analytes such as urea, insulin, calcium, and cardiac biomarkers [70,71,72,73,74,75]. Herein, this study is the synthesis of copper-based optical material using low-temperature-assisted plasma. Hydrogen (H_2_) gas will be used during plasma treatment and will act as a reducing agent [76]. The study also reports the manipulation of this material as an optical sensor for the detection of ovarian cancer marker CA 125.

## 2. Materials and Methods

All chemicals used in this study were used as such unless stated otherwise. Copper chloride (ACROS ORGANICS, 99%) and pyrrole (TGI, 99%) were obtained from Fischer Scientific USA (Waltham, MA, USA). Hydrogen gas was obtained from Airgas (Radnor, PA, USA). CA 125 antibody was obtained from Biosynth (Gardner, MA, USA). Different concentrations of CA 125 antigen are obtained from United Immunoassay (San Francisco, CA, USA).

### 2.1. Methods

The absorbance of the synthesized optical material and antibody conjugated optical material were recorded using UV-Vis-NIR double beam absorbance spectrophotometer (LAMBDA 950, PerkinElmer, Shelton, CT, USA) by dispersing in solvent. The spectra were recorded using 1 cm path length quartz cuvette.

The chemical state and elemental composition of the synthesized optical materials were confirmed using an X-ray photoelectron spectroscopy (XPS). The spectra were recorded using the Physical Electronics Phi 5000 VersaProbe (Chanhassen, MN, USA). The powdered samples were used for the identification of the compound. Both survey and high-resolution spectra were recorded for the analysis of the synthesized copper nanoparticles.

The functional groups present in the copper nanoparticles were confirmed using Fourier transform infrared spectroscopy (FTIR). The Bruker FTIR spectrometer (Billerica, MA, USA) in ATR mode with 120 scans with wavenumbers ranging from 4000 cm^−1^ to 400 cm^−1^ was used for recording the spectra. The zeta potential of the synthesized optical material was measured by electrophoretic light scattering (25 °C; disposable folded capillary cell (DTS1070); 173°; *n* = 3 per batch) using the Zetasizer Nano ZS (Malvern Panalytical, Westborough, MA, USA).

### 2.2. Synthesis of Optical Material of Copper Pyrrole (CuPy)

The CuPy material was synthesized using radio-frequency-assisted low-temperature plasma. Before synthesis, the plasma chamber was cleaned using radio frequency (RF) plasma without any reagents or gases. First, stock solution was prepared by dissolving 15 mg of copper chloride in 3 mL of pyrrole. The color of the solution turned green after dissolving in pyrrole. An amount of 1 mL from the stock solution was placed inside the chamber in a 5 mL glass bottle. Once the pressure dropped below 1 Torr, the RF was switched on in the low-temperature plasma (Harrick Plasma, Plasma Cleaner PDC–001–HP, Ithaca, NY, USA) for 10 min. After 10 min of plasma generation of the precursor in the chamber, H_2_ gas was turned on with a flow rate of 50 sccm for another 10 min. The products were formed inside the plasma chamber. Two types of products were extracted from the chamber (one in water and another in methanol). Additionally, a separate pyrrole-only material was created using 1 mL of pyrrole. This sample was processed identically to the copper and pyrrole solution. All products were analyzed using UV–visible absorbance spectroscopy, fluorescence spectroscopy, XPS, and FTIR. Zeta potential measurements were performed on the base sensor material before and after modification with the antibody.

### 2.3. Synthesis of CA 125 Sensor Using CuPy (AbCuPy)

The sensor was developed by incubating CuPy with the CA 125 antibody. The stock solution of CA 125 had a concentration of 1 mg/mL. The working solution was made by mixing 20 μL of antibody solution and 980 μL of water. The methanol-extracted CuPy was stirred with 200 μL of CA 125 antibody working solution for 12 h. After 12 h, the material was washed with water and extracted in methanol for the detection of CA 125.

### 2.4. Detection of CA 125 Using AbCuPy

**Figure 1:**
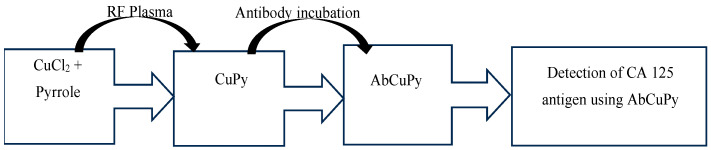
Synthesis of the antibody-modified CuPy sensor.

CA 125 antigens of different concentrations (25 U/mL, 75 U/mL, 100 U/mL, 150 U/mL, and 300 U/mL) were used for monitoring the sensing efficacy of the developed sensor. A total of 5 µL from each concentration was added to 3 mL of CuPy modified with CA 125 antibody (AbCuPy) and monitored for the change in fluorescence emission of AbCuPy (manufacturer’s recommended dilution). Figure 1 shows a schematic of the process for synthesizing the antibody-modified CuPy sensor.

## 3. Results and Discussion

### 3.1. Synthesis and Characterization Photonic Material of Copper Using Low-Temperature Plasma

**Figure 2:**
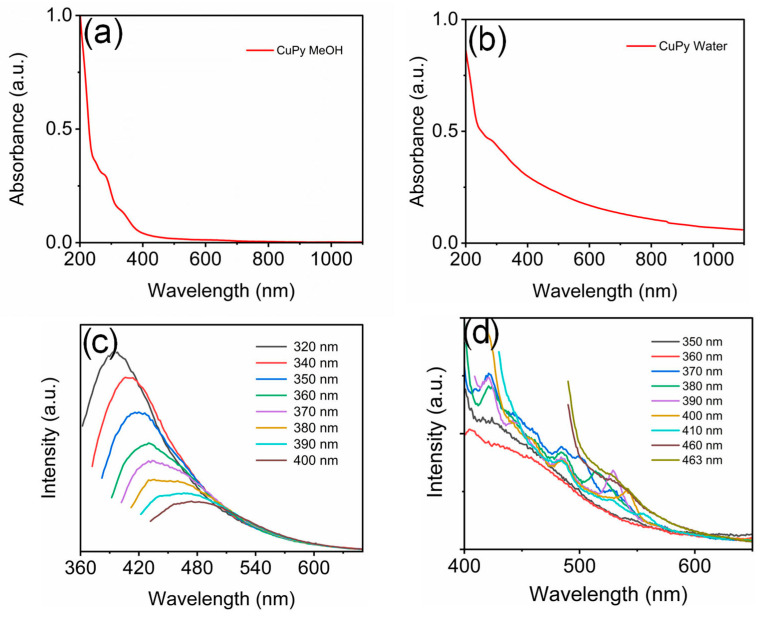
The UV–visible absorbance spectra: (**a**) CuPy product extracted in methanol; (**b**) CuPy product extracted in water; (**c**) fluorescence spectra of CuPy product extracted in methanol; (**d**) fluorescence spectra of CuPy product extracted in water.

The plasma-assisted method is employed for the synthesis of novel optical material of Cu using pyrrole. Apart from its volatile nature, pyrrole is known to react with Cu. The nitrogen atom in pyrrole acts as a nucleophile and allows it to form a coordinate bond with the electrophilic Cu ion [77,78,79]. The dynamic protonation of the pyrrole group is also favorable for the formation of a new material. The exact chemical composition and its fundamental aspects are not included in this study, but the characterization methods used give excellent structural information. Here, we mainly focus on the utilization of nonthermal plasma for the creation of optical materials of Cu and its application in the field of biosensors. Plasma processing of 1 mL of solution produced 2–3 mg of material. Two different solvents, methanol and water, were used to extract the material, but neither solvent produced complete dissolution. Therefore, the use of low-temperature plasma for the Cu and pyrrole solution extracted two products with one soluble in water and one in methanol. Figure 2 shows the UV–visible absorbance spectra and fluorescence spectra of the synthesized products. The pyrrole-only solution produced one product soluble in water.

From Figure 2, it is observed that the UV–visible absorbance spectrum of methanol-extracted CuPy shows absorbances around 251 nm, 282 nm, and 339 nm. The absorbance around 251 nm can be attributed to the pyrrole to Cu (ligand to metal) charge transfer [80]. The electronic transition around 282 nm and 339 nm may be due to the combined effect of d-d transition within the Cu ion and ligand to metal charge transfer [80,81]. As a result of these complex electronic interactions, these transitions are slightly broader. From the electronic spectra, one cannot completely rule out the possibility of pyrrole oligomers like bipyrrole and terpyrrole [81,82]. This spectrum is different than the water-extracted product. The UV–visible absorbance spectrum of water-extracted CuPy shows different electronic transitions compared to methanol-extracted product. It shows broader absorbance characteristics similar to the pyrrole-only material in water (Figure 2b and Figure S1) with a shift. As there are several similarities between the pyrrole-only material and water-extracted CuPy, further characterization focused on examining the differences between methanol-extracted and water-extracted CuPy. Figure 2c,d show the fluorescence emission of methanol- and water-extracted products. The methanol-extracted product shows distinct molecular emission characteristics in the visible region of the electromagnetic spectrum when compared to water-extracted product. Moreover, the methanol-extracted product shows red edge excitation shift in the fluorescence emission. The exact reason was not clear, and plausible reasons can be the ligand to metal charge transfer through the nitrogen atom of pyrrole to Cu, solvent dynamics, internal vibrational relaxation due to electron phonon coupling, or aggregation-induced emission [83]. The corresponding excitation of CuPy in both water and methanol is shown in Figure S2.

**Figure 3:**
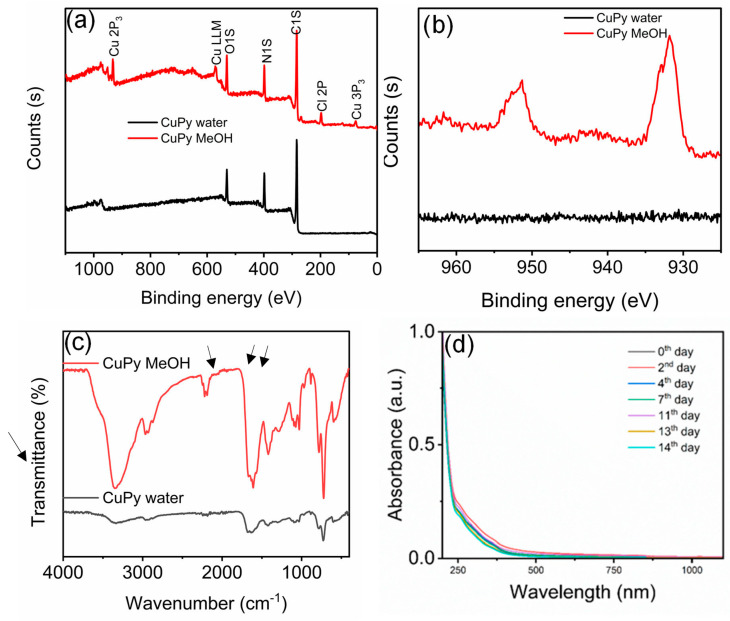
XPS spectra showing the binding energy of CuPy in methanol extract and CuPy in water extract: (**a**) survey spectra; (**b**) high-resolution spectra of Cu; (**c**) FTIR spectra of CuPy; (**d**) stability of CuPy in methanol.

The methanol-extracted and water-extracted CuPy materials were further analyzed using XPS to evaluate its elemental composition and oxidation state. The survey spectrum (Figure 3a) clearly reveals the presence of copper and nitrogen (N_2_) and confirms that both copper and pyrrole are present in the methanol-extracted CuPy complex. The percentages of Cu and N_2_ in the methanol-extracted CuPy are 2% and 14%, respectively. Interestingly, the water-extracted CuPy does not show the presene of copper. The presence of nitrogen implies the water-extracted product contains pyrrole. From the high-resolution spectra (Figure 3b) of Cu, the presence of satellite peak around 941.71 eV confirms the 2+ oxidation of Cu. This confirmation of the oxidation state correlates to the origin of d-d transition in the electronic spectra around 282 nm.

From the FTIR spectra (Figure 3c and Figure S3) the N-H bending vibrations of pyrrole normally seen at 3398 cm^−1^ broadened and shifted to 3330.23 cm^−1^ (identified in figure) [80]. This can be due to the binding or coordination of Cu with pyrrole’s nitrogen moiety which affects the electron density around nitrogen. Similarly, new vibrational modes were observed in the FTIR spectra around 781.02 cm ^−1^ and 878.23 cm ^−1^ (identified in figure). This might be the characteristic vibrations of Cu-N stretching or Cu-H stretching or due to other metal ligand-related vibrations of Cu and pyrrole [84]. The change in the vibrational modes of C-N of pyrrole from 1075.59 cm^−1^, 1045.60 cm^−1^, and 1012.74 cm^−1^ to 1098.89 cm^−1^, 1075.33 cm^−1^, and 1026.47 cm^−1^ confirms the binding of Cu with pyrrole (identified in figure) [80,85]. The aromatic C-H bending vibrations of pyrrole shifted to 596.83 cm^−1^ and 724.90 cm^−1^ from 543.87 cm^−1^ and 731.22 cm^−1^, respectively, after Cu and plasma treatment [86].

The stability of the methanol-extracted CuPy was monitored using UV–visible absorbance spectroscopy for up to 14 days in solution. CuPy stock solution was prepared and kept at 4 °C. The material shows excellent stability in solution for up to 14 days (Figure 3d). From 0 to 14 days, the characteristic absorbance features remain intact. This indicates that the CuPy remains as such without any further degradation or aggregation. This monitoring shows the solution phase stability of the synthesized CuPy.

The stability of the methanol-extracted CuPy was also assessed through a range of pH values to evaluate its functionality and reliability by varying acidic and basic conditions. The UV–visible absorbance spectra and fluorescence emission spectra of CuPy at different pH values demonstrated excellent stability which indicates that the product retains its optical properties across a wide pH range (as shown in Figure S4). The fluorescence emission of CuPy at different pH levels was monitored by exciting at 390 nm. The fluorescence emission spectra did not show any change in the emission wavelength for all pH levels (Figure S4b). However, the emission intensity changed slightly due to the change in local environment as observed in UV–visible absorbance spectra. This study shows that the CuPy is optically stable with minimal changes in fluorescence emission at pH 10.

### 3.2. Development of CA 125 Sensor Using CuPy

A selective detection CA 125 sensor was developed by conjugating methanol-extracted CuPy with CA 125 antibody. The AbCuPy will be selective for the tumor marker CA 125. The CuPy was electrostatically modified with CA 125 antibody for 12 h.

**Figure 4:**
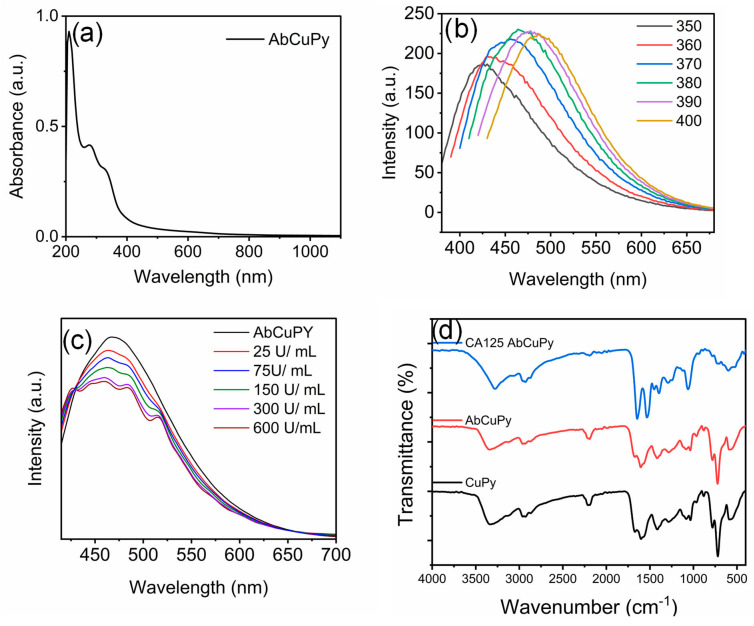
(**a**) UV–visible absorbance spectra of AbCuPy: (**b**) fluorescence emission of AbCuPy at different excitation; (**c**) sensing of AbCuPy; and (**d**) FTIR spectra of AbCuPy with 600 U/ mL CA 125 added.

From Figure 4a, the UV–visible spectrum showed that antibody conjugation resulted in the appearance of a new peak around 200 nm and signifies the modification. Additionally, the peak at 340 nm is shifted to 330 nm. These changes in the electronic spectra of antibody conjugated CuPy are indicators of CA 125 antibody functionalization. Furthermore, the antibody binding with CuPy was further monitored using FTIR spectroscopy. From Figure 4d, AbCuPy retains all the characteristic vibrations of CuPy with a slight shift of ±3 cm^−1^ to 5 cm^−1^. This shift can be attributed to the binding of CA125 antibody with CuPy.

Figure 4b shows the excitation-dependent emission of CuPy after modification with CA 125 antibody. Unlike CuPy, antibody binding resulted in the emission shifts towards higher wavelengths, but its intensity increased. This observation can be explained based on aggregation-induced emission, a photophysical phenomenon observed in certain fluorophores or metal-enhanced emissions [87,88]. This phenomenon may originate due to the binding of CA 125 antibody with CuPy and results in the decreased interactions between individual particles when compared to CuPy. This aggregation with metal is due to molecular packing and leads to a red-shifted emission (shift to longer wavelengths) with an increase in fluorescence intensity. This result contrasts with the usual fluorescence quenching observed in many molecular systems upon aggregation. This type of assembly-induced emission has been reported in copper materials [89,90]. The zeta potential of methanol-extracted CuPy and CA 125-modified CuPy were found to be +5.07 mV and −18.45 mV, respectively (Figure S5). These measurements also confirm the modification of the sensor with the antibody [91,92,93].

The sensing efficacy of CA 125-modified CuPy was monitored by adding 5 μL of CA 125 antigen from 25 U/mL, 75 U/mL, 150 U/mL, 300 U/mL, and 600 U/mL stock solutions. The standard level of CA 125 antigen regarded as a positive test is a reading above 35 U/mL [44]. This level is accepted as positive for both serological and tissue samples and with different radioimmunoassay tests [44,94,95]. As mentioned previously, optical sensors are designed to towards a target analyte. Binding with this analyte changes the optical properties of the sensors. With this sensor being an optical sensor (fluorescence-based), the samples were monitored for a decrease in fluorescence intensity due to the binding of the antigen to AbCuPy [96]. The antigen binding resulted in a decrease in fluorescence intensity for all samples tested. Higher concentrations of antigen resulted in the appearance of shoulder peak around 517 nm. This decrease in fluorescence intensity can be used for the monitoring of CA 125 from an unknown sample. From the FTIR, it is observed that CA 125 antigen binding resulted in the change in the vibrations of AbCuPy (Figure 3d).

The selectivity of CA 125-antibody-modified CuPy towards CA 125 was tested against different species, amino acids, etc., in Figure S5. The sensor is found to be selective for CA 125 compared with other analytes.

## 4. Conclusions

In summary, a new technique was developed to synthesize an optically active material containing Cu using low-temperature plasma. The synthesized copper-based material was found to have optical features. It showed a red edge excitation emission in the visible region of the electromagnetic spectrum. From the XPS spectra, CuPy was found to contain both Cu and pyrrole. Additionally, the developed CuPy was found to be stable for up to 14 days. The UV–visible absorbance spectra of CuPy at different pH levels showed that CuPy was highly stable across a broad range of pH levels. CA 125 antibody conjugation resulted in the enhancement of the fluorescence intensity due to the aggregation-induced emission. The detection of CA 125 resulted in the concentration-dependent fluorescent quenching of CA 125-antibody-modified CuPy. The developed sensor is found to be selective for CA 125. To conclude, the fast and scalable synthesis of a metal-based optical material was developed using low-temperature plasma, and it was found to be effective for the detection of CA 125 ovarian cancer marker. The future goal of the study is to apply low-temperature plasma to the fabrication of different optical materials by changing the metal and organic precursor along with molecular simulation and TDFT calculations to identify the geometry. The efficacy of CuPy will be studied further using clinical samples.

## Data Availability

All data are presented in the manuscript and in Supplementary Information.

## Supplementary Materials

The following supporting information can be downloaded at: https://www.mdpi.com/article/10.3390/nano16140894/s1. Figure S1: UV-visible absorbance spectra of pure pyrrole in water; Figure S2: Excitation spectra of (a) CuPy methanol extract and (b) CuPy water extract; Figure S3: FTIR spectra of (a) pure pyrrole (b) overlayed with CuPy in methanol extract and CuPy in water extract; Figure S4: (a) UV-visible absorbance spectra of CuPy (b) Emission spectra of CuPy at different pH; Figure S5: UV-visible absorbance spectra of AbCuPy upon interaction with different analytes.
