# Supplementary material for: Plasma-Corona Enabled Synthesis of Photonic Copper Sensor for the Detection of Ovarian Cancer Marker CA 125
Source: Nanomaterials (Basel). 2026 Jul 21;16(14):894. doi: 10.3390/nano16140894 (PMC13415857; doi:10.3390/nano16140894)
Supplement: Supplementary file 1 [file nanomaterials-16-00894-s001.zip › nanomaterials-4414391-supplementary.pdf]

Plasma-Corona Enabled Synthesis of Photonic Copper Sensor for the Detection of Ovarian Cancer Marker CA 125

Kimberly M. Jones [1,†], Takumi Uesaka [1,†], Lakshmi V. Nair [1,2] and Vinoy Thomas [1,3,4,*]

[1] Department of Mechanical and Materials Engineering, School of Engineering, University of Alabama at Birmingham, Birmingham, AL 35294, USA; tuesaka@uab.edu (T.U.); lakshmi.vnair@vit.ac.in (L.V.N.)
[2] Centre for NanoBiotechnology, Vellore Institute of Technology, Vellore 632014, Tamil Nadu, India
[3] Center for Nanoscale Materials and Biointegration (CNMB), University of Alabama at Birmingham, Birmingham, AL 35294, USA
[4] Center for Clinical and Translational Sciences (CCTS), University of Alabama at Birmingham, Birmingham, AL 35294, USA
[*] Correspondence: vthomas@uab.edu
[†] These authors contributed equally to this work.

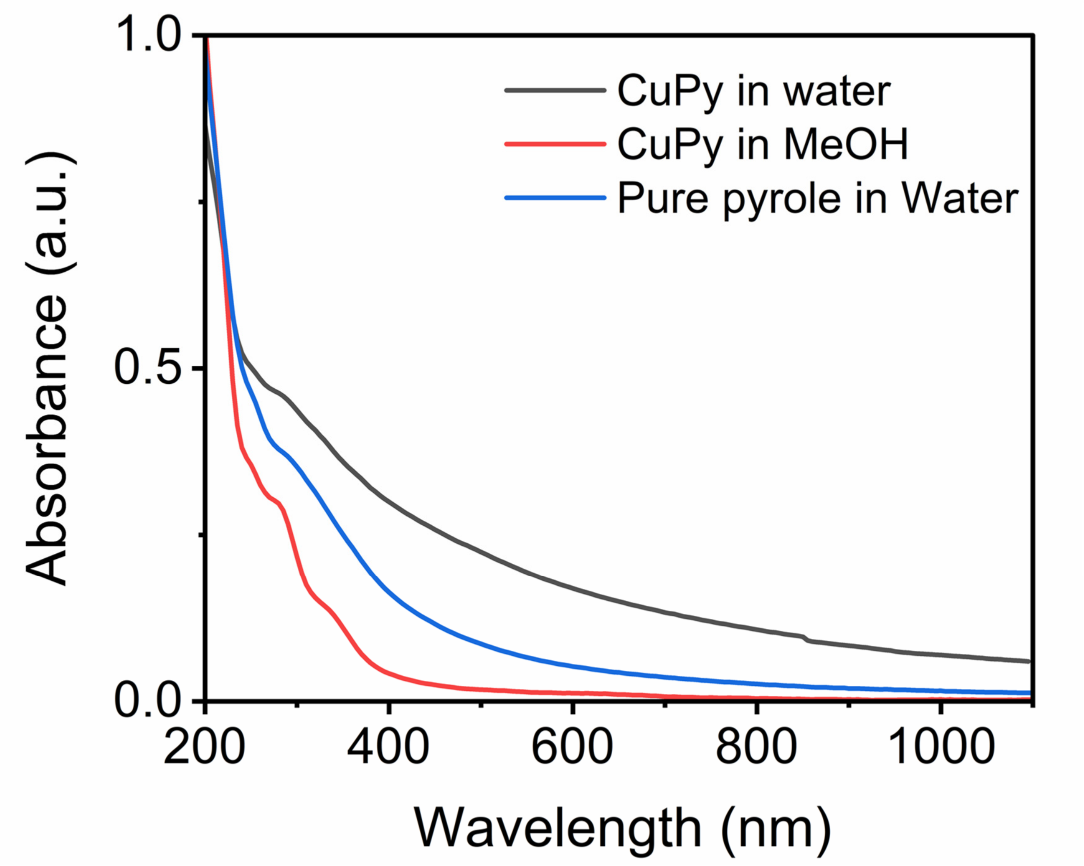

Figure S1: UV-visible absorbance spectra of pure pyrrole in water

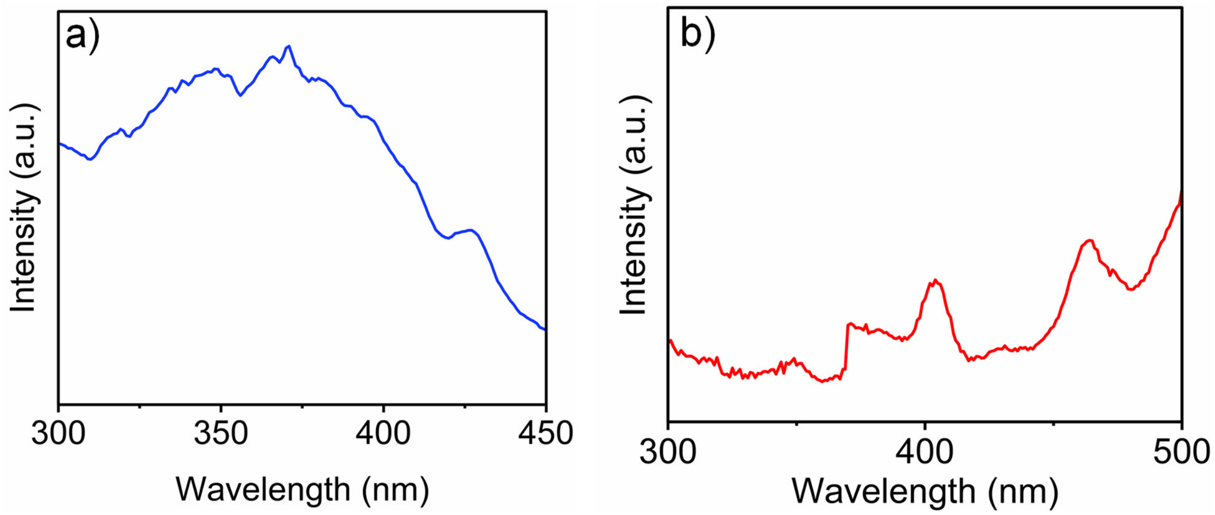

Figure S2: Excitation spectra of a) CuPy methanol extract and b) CuPy water extract.

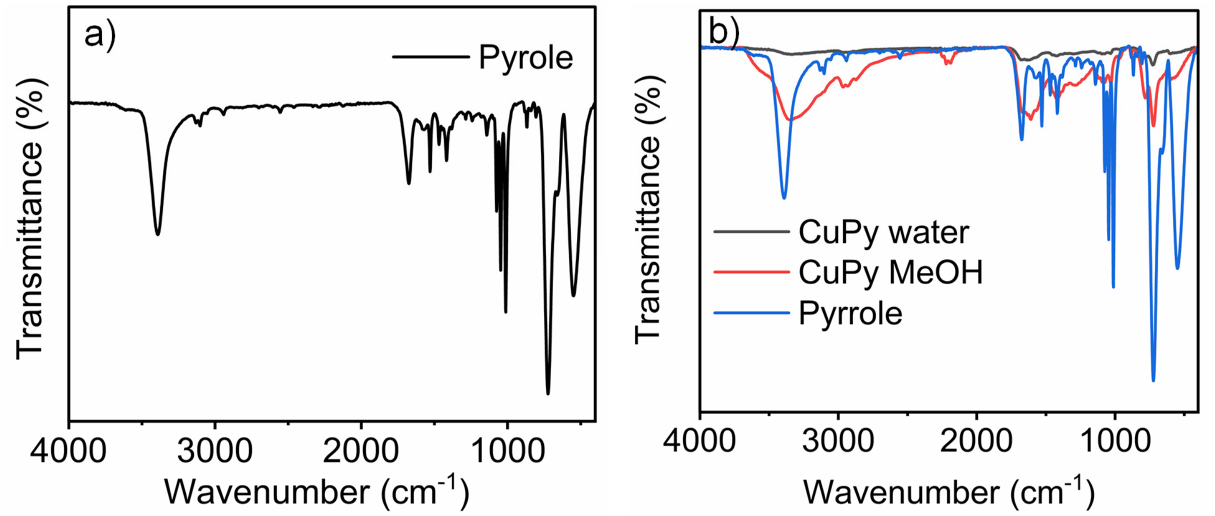

Figure S3: FTIR spectra of a) pure pyrrole b) overlayed with CuPy in methanol extract and CuPy in water extract.

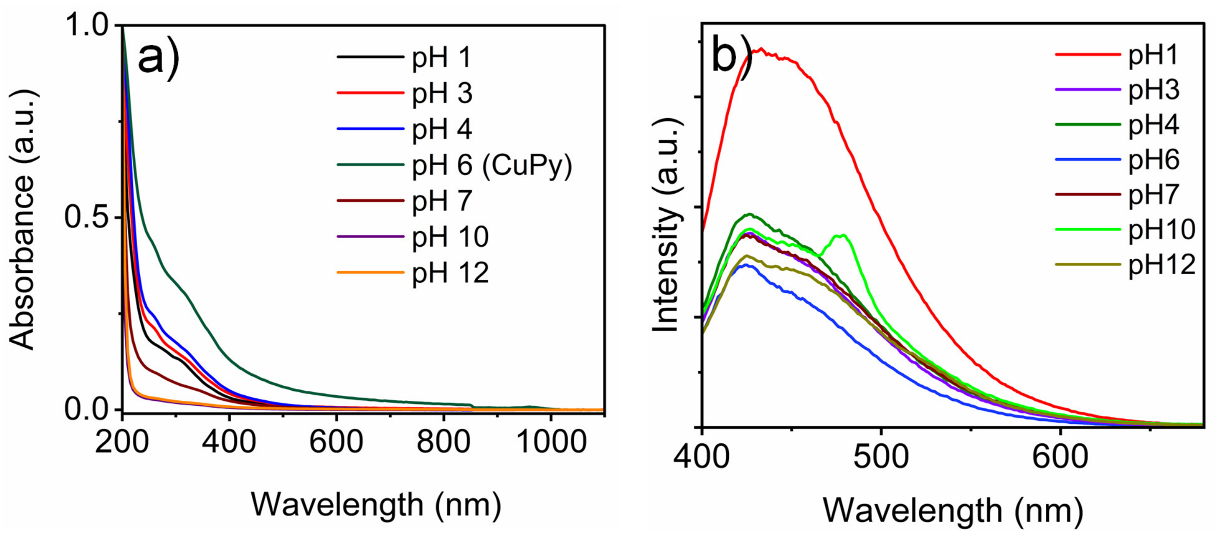

Figure S4: a) UV-visible absorbance spectra of CuPy b) Emission spectra of CuPy at different pH

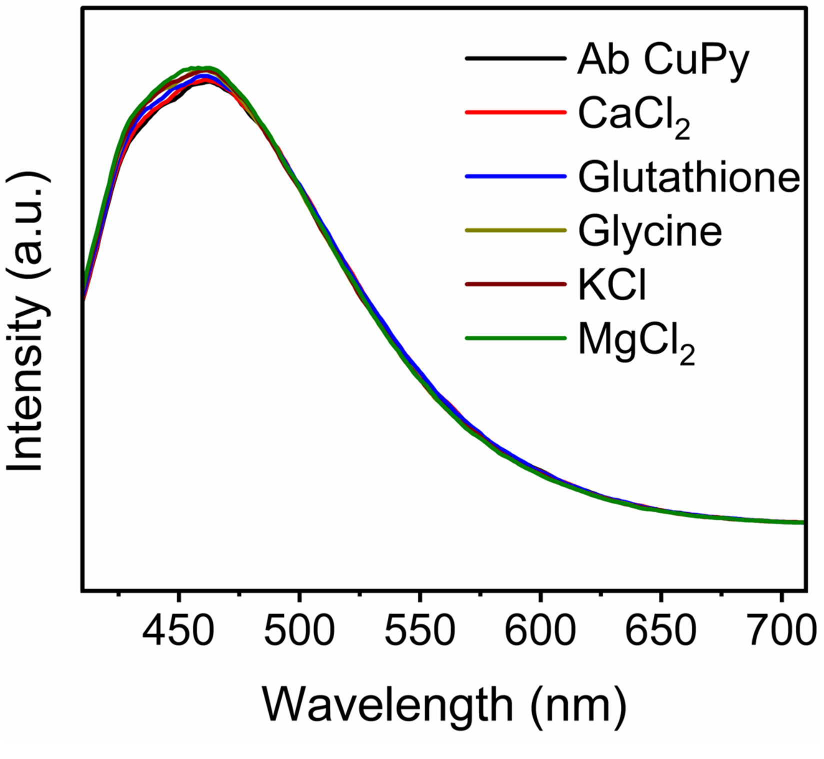

Figure S5: UV-visible absorbance spectra of AbCuPy upon interaction with different analytes
